# Increased Mortality and Healthcare Costs Upon Hospital Readmissions of Ulcerative Colitis Flares: A Large Population-Based Cohort Study

**DOI:** 10.1093/crocol/otab029

**Published:** 2021-06-09

**Authors:** Simcha Weissman, Sachit Sharma,, Brian M Fung, Muhammad Aziz, Michael Sciarra, Arun Swaminath, Joseph D Feuerstein

**Affiliations:** 1 Department of Medicine, Hackensack Meridian Health Palisades Medical Center, North Bergen, New Jersey, USA; 2 Department of Medicine, University of Toledo Medical Center, Toledo, Ohio, USA; 3 Division of Gastroenterology and Hepatology, University of Arizona College of Medicine—Phoenix, Phoenix, Arizona, USA; 4 Division of Gastroenterology, University of Toledo Medical Center, Toledo, Ohio, USA; 5 Department of Gastroenterology and Hepatology, Hackensack Meridian Health Palisades Medical Center, North Bergen, New Jersey, USA; 6 Division of Gastroenterology, Inflammatory Bowel Disease Program, Lenox Hill Hospital, New York, New York, USA; 7 Division of Gastroenterology, Beth Israel Deaconess Medical Center, Harvard Medical School, Boston, Massachusetts, USA

**Keywords:** ulcerative colitis, inflammatory bowel disease, readmission, predictors, mortality

## Abstract

**Background:**

Ulcerative colitis (UC) flares often result in prolonged hospitalization and considerable mortality. Nevertheless, large-scale analyses evaluating the frequency and characteristics of hospital readmissions for UC remain limited. We aimed to examine these clinical outcomes in a nationwide cohort of patients hospitalized with UC.

**Methods:**

We queried the 2017 Nationwide Readmission Database using ICD-10-CM codes to identify all adult patients admitted for UC. Outcomes including mortality, readmission rates, predictors of readmission and mortality, and healthcare usage were assessed. Multivariate analysis was used to adjust for potential confounders.

**Results:**

From the 31,063 patients hospitalized for UC, 17.38% were readmitted within 30 days and 28.51% in 90 days. UC accounted for 28.17% and 29.82% of readmissions at 30 and 90 days, respectively. Compared to index admission, 30- and 90-day readmissions were characterized by significantly higher mortality (0.42% vs 1.99% and 1.65%, respectively), longer hospital stays (5.05 vs 6.62 and 6.04 days, respectively), and increased hospital cost ($49,999 vs $62,288 and $59,698, respectively) (all *P* < 0.01). Numerous factors, including chronic steroid use [hazard ratio (HR) 1.35] and opioid use (HR 1.6, were independently associated with increased 30-day readmission (*P* < 0.01). Numerous factors, including anxiety (HR 1.21) and venous thromboembolism (HR 5.39), were independently associated with increased 30-day mortality (*P* < 0.01).

**Conclusions:**

In a large cohort of patients hospitalized for UC, we found that readmission is associated with higher mortality and more lengthy/costly admissions. Additionally, we found independent associations for readmission and mortality that may help identify patients who can benefit from close postdischarge follow-up.

## Introduction

Inflammatory bowel disease (IBD) is a chronic inflammatory disorder mainly comprised of Crohn disease and ulcerative colitis (UC). During flares, symptoms can be severe enough to require hospitalization. In fact, it is estimated that 18%–25% of patients with UC require at least 1 hospitalization for UC during their lifespan.^1^ This, in tandem with an increasing worldwide incidence and prevalence of IBD, poses a substantial burden on healthcare resources.^2–4^

Despite improved and newer therapeutic modalities for the management of patients with IBD, acute UC flares often result in prolonged hospitilization and subsequently pose a risk of mortality.^5^ While the mortality rate in patients with IBD is generally thought to be low, large population-based studies evaluating inpatient mortality—particularly in regards to patients who are readmitted—is lacking.^5,6^ One could postulate that the risk of mortality may be higher in the subset of patients requiring hospital readmission. Indeed, most prior data on UC readmissions have been from small study samples, case series, and single-center experiences.^7–11^

Herein, we aimed to perform a large, population-based study to identify rates of mortality and healthcare usage, as well as their predictors, in patients admitted for UC.

## Methods

### Data Source

In this retrospective cohort study, we queried the National Readmission Database (NRD) from January 2017 to November 2017. The NRD is the largest database of hospital readmissions in the United States and is derived from billing data submitted to statewide organizations based upon discharge abstracts. The NRD 2017 is nationally representative as it contains data from almost 18 million hospital stays (unweighted) in 2454 hospitals in 28 states, accounting for almost 60% of the US population. It contains deidentified clinical and nonclinical elements at both the patient- and hospital level using the International Classification of Diseases-10th revision (ICD-10) coding system.

### Study Population

**Figure 1.**
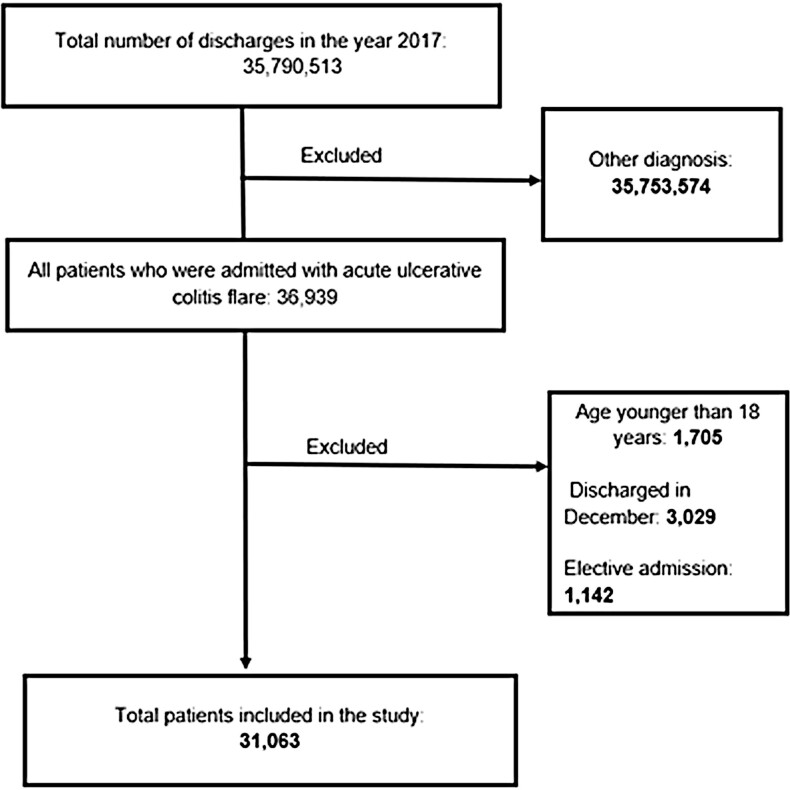
Flow diagram illustrating study inclusion and exclusion criteria.

Patients with a primary diagnosis of UC during hospitalization (based on ICD-10 diagnostic codes K51.90 and K51.91) were included in the study. Patients were excluded if: (1) they were less than 18 years of age, (2) they were admitted electively, or (3) they were admitted within 30 days of the end of the fiscal year (as all patients were followed for at least 30 days upon discharge) (Fig. 1). All-cause mortality, length of stay (LOS), and cost during the index hospital admission were identified. Thereafter, these patients (who were discharged) in January of 2017 were followed until November 2017 to determine the percentage readmitted within 30 and 90 days. All-cause hospitalization mortality, LOS, and cost were again identified, now upon readmission. Patient demographic, hospital demographic, and other risk factors (based on ICD-10 codes) for readmission and mortality, along with the most common diagnoses/causes of readmission, were also recorded. We reviewed prior studies to help select and categorize diagnostic codes that would accurately identify patients with UC.^12–14^Supplementary Table 1 for the ICD-10 codes used in the study.

### Ethical Considerations

Institutional Review Board approval was not required for this study as it was performed using deidentified and nationally available data.

### Study Variables

Patient demographics included age, sex, median household income, primary expected payer, and burden of comorbidities, assessed by the Charlson Comorbidity Index (CCI). Hospital demographics included hospital size (based on the number of beds), teaching status, region, and location.

### Primary and Secondary Outcomes

The primary outcome was mortality rate during (1) index admission, (2) 30-, and (3) 90-day readmission. Secondary outcomes were: (1) rates of readmission, (2) causes for readmission, (3) risk factors associated with readmission and mortality, as well as (4) mean hospitalization LOS and costs.

### Statistical Analysis

Statistical analyses were performed using STATA, version 16.1 (StataCorp, College Station, TX). Weighting of patient-level observations was implemented. Univariate analysis was initially performed to calculate unadjusted odds ratio and determine confounders significantly associated with the outcomes. Multivariate regression analysis was used to adjust for potential confounders. A multivariate regression model was then built by including all confounders that were found to be significant by univariate analysis, to calculate an adjusted odds ratio. Logistic regression was used for binary outcomes and linear regression was used for continuous outcomes. Proportions were compared using Fisher exact test, and continuous variables were compared using Student *t* test. All *P* values were 2-sided, with 0.05 as the threshold for statistical significance.

## Results

### Baseline Patient and Hospital Characteristics

A total of 31,063 adult patients with a principal diagnosis of UC (upon discharge) were included in the study. The mean age was 48.8 years, the majority of patients were female (53.79%), and Medicare (48.1%) was the primary payer insurance. Patients were predominantly admitted to large (56.5%) teaching hospitals (71.04%) in large metropolitan areas (61.3%) (Table 1).

**Table 1.** Baseline Hospital- and Patient-Level Characteristics of Those Admitted for a UC Flare

Variable	N (%)
Total population	31,063
Female	16,709 (53.79%)
Mean age in years	48.8
Insurance provider	
Medicare	9269 (29.84%)
Medicaid	5156 (16.6%)
Private	14,957 (48.15%)
Uninsured	1681 (5.38%)
CCI	
0	19,197 (61.80%)
1	6293 (20.26%)
2	2494 (8.03%)
3 or more	3079 (9.91%)
Median income in patient zip code	
0th to 25th percentile	7887 (25.39%)
26th to 50th percentile	8101 (26.08%)
51st to 75th percentile	7887 (25.39%)
76th to 100th percentile	7188 (23.14%)
Hospital location	
Large metropolitan with at least 1 million residents	19,048 (61.32%)
Small metropolitan with less than 1 million residents	9940 (32.00%)
Micropolitan areas	1541 (4.96%)
Nonmetropolitan or micropolitan	534 (1.72%)
Hospital size	
Small	5184 (16.69%)
Medium	8325 (26.80%)
Large	17,554 (56.51%)
Teaching hospital	22,067 (71.04%)

### Mortality Rates Upon Index Admission and Readmission

**Figure 2.**
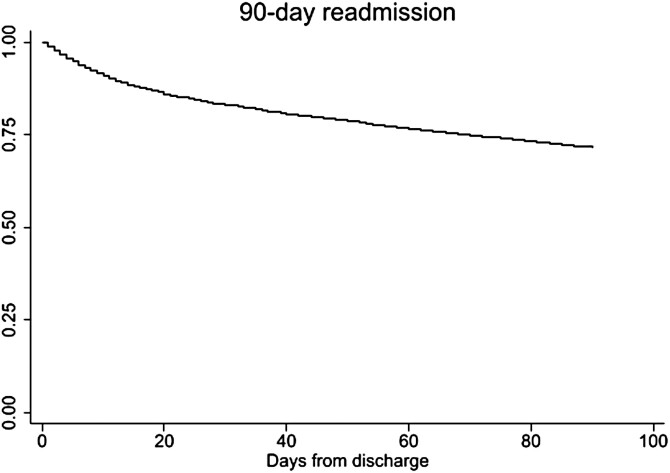
Graph depicting the trend of mortality upon readmission after hospitalization for a UC flare.

Upon multivariate analysis, when compared to mortality on index admission, mortality upon 30- and 90-day readmission was significantly higher (0.42% vs 1.99% and 1.65%, respectively, *P* < 0.01) (Fig. 2). Upon sensitivity analysis, to exclude the possibility of higher acuity on readmission (in general) as driving the mortality rates, we found that the 1708 patients readmitted for UC had a higher overall mortality (2.29% vs 1.87%, *P* < 0.05) as compared to the other (3663) readmitted patients.

### Healthcare Use Upon Index Admission and Readmission

Upon multivariate analysis, when compared to the index admission, 30-day readmissions were characterized by significantly longer mean LOS (5.05 vs 6.62 days, *P* < 0.01) and higher mean hospitalization charges ($49,999 vs $62,288, *P* < 0.01). As an aggregate, 30-day readmission accounted for an additional 35,481 inpatient days and $334 million in hospitalization cost nationally.

Upon multivariate analysis, when compared to the original index admission, 90-day readmissions were also characterized by significantly longer mean LOS (5.04 vs 6.04 days, *P* < 0.01), and higher mean hospitalization charges ($49,036 vs $59,698, *P* < 0.01).

### Readmission Rates and Reasons for Readmission

Of the 30,903 patients discharged from their index hospitalization and followed for 30 days, 17.38% (5371) were readmitted (within 30 days). Of the 24,553 patients discharged from the index hospitalization and followed for 90 days, 28.51% (7001) were readmitted (within 90 days).

UC was the most common primary diagnosis at both 30- (31.8%) and 90-day (31.25%) readmission. Table 2 contains a list of the most common primary diagnoses at 30- and 90-day readmission.

**Table 2.** Most Common Primary Diagnosis During 30- and 90-Day Readmission After Hospitalization for a UC Flare

S. No.	Primary Diagnosis at 30-Day Readmission	N (%)
1	“Ulcerative colitis, unspecified, without complication”	720 (13.40%)
2	“Ulcerative colitis, unspecified with rectal bleeding”	374 (6.97%)
3	“Sepsis, unspecified organism”	302 (5.62%)
4	“Ulcerative (chronic) pancolitis with rectal bleeding”	213 (3.96%)
5	“Ulcerative (chronic) pancolitis without complications”	206 (3.84%)
S. No.	Primary Diagnosis at 90-Day Readmission	N (%)
1	“Ulcerative colitis, unspecified, without complication”	900 (12.85%)
2	“Encounter for attention to ileostomy due to colitis”	509 (7.27%)
3	“Ulcerative colitis, unspecified with rectal bleeding”	421 (6.02%)
4	“Sepsis, unspecified organism”	372 (5.32%)
5	“Ulcerative (chronic) pancolitis without complications”	258 (3.68%)

### Risk Factors Associated With Readmission

Upon multivariate analysis, higher CCI [hazard ratio (HR) 1.08, *P* < 0.01], longer LOS (HR 1.01, *P* < 0.01), chronic steroid use (HR 1.35, *P* < 0.01), admission to a teaching hospital (HR 1.12, *P* < 0.05), opioid use (HR 1.65, *P* < 0.01), venous thromboembolism (VTE) (HR 1.35, *P* < 0.01), and congestive heart failure (HR 1.34, *P* < 0.01) were independently associated with increased 30-day readmission. In contrast, female sex (HR 0.90, *P* < 0.05) was not associated with increased 30-day readmission.

Upon multivariate analysis, higher CCI (HR 1.09, *P* < 0.01), longer LOS (HR 1.01, *P* < 0.01), anxiety (HR 1.21, *P* < 0.01), opioid use (HR 1.52, *P* < 0.01), admission to a teaching hospital (HR 1.18, *P* < 0.01), and discharge to other than home (HR 1.27, *P* < 0.01) were independently associated with increased 90-day readmission rate. In contrast, older age (>65) (HR 0.99, *P* < 0.01) was not associated with increased 90-day readmission. Table 3 for the risk factors independently associated with readmission.

**Table 3.** The numerous risk factors that were found to be associated with 30- and 90-day readmission in patients admitted for ulcerative colitis (UC) flares

Risk Factors for 30-Day Readmission	HR	*P*
CCI score	1.08	**<0.01**
LOS	1.01	**<0.01**
Chronic steroid use	1.35	**<0.01**
Teaching hospital status	1.12	**<0.05**
Opioid use	1.65	**<0.05**
VTE	1.35	**<0.05**
Congestive heart failure (CHF)	1.34	**<0.05**
Female sex	0.90	**<0.05**
Risk Factors for 90-Day Readmission	HR	*P*
CCI score	1.09	**<0.01**
LOS	1.01	**<0.01**
Anxiety	1.21	**<0.01**
Opioid use	1.52	**<0.01**
Teaching hospital status	1.18	**<0.01**
Discharge to other than home	1.27	**<0.01**
Older age (>65)	0.99	**<0.01**

Bold text highlights statistical significance.

### Risk Factors Associated With Mortality

Upon multivariate analysis, longer LOS (HR 1.01, *P* < 0.01), higher CCI (HR 1.35, *P* < 0.01), anxiety (HR 1.21, *P* < 0.01), VTE (HR 5.39, *P* < 0.01), acute kidney injury (HR 4.92, *P* < 0.01), opioid use (HR 1.52, *P* < 0.05), admission to a teaching hospital (HR 1.18, *P* < 0.05), and discharge to other than home (HR 1.18, *P* < 0.05) were independently associated with increased 30-day mortality. In contrast, older age (HR 0.99, *P* < 0.05), was not associated with increased 30-day mortality.

Upon multivariate analysis, older age (HR 1.05, *P* < 0.01), higher CCI (HR 1.46, *P* < 0.01), end-stage renal disease (HR 3.62, *P* < 0.05), and acute kidney injury (HR 4.92, *P* < 0.01) were independently associated with increased 90-day mortality. Table 4 for the risk factors independently associated with mortality.

**Table 4.** The numerous risk factors that were found to be associated with 30- and 90-day mortality in patients admitted for ulcerative colitis (UC) flares

Risk Factors for 30-Day Mortality	HR	*P*
CCI score	1.09	**<0.01**
LOS	1.01	**<0.01**
Anxiety	1.21	**<0.01**
VTE	5.39	**<0.01**
Acute kidney injury (AKI)	4.92	**<0.01**
Teaching hospital status	1.18	**<0.05**
Opioid use	1.52	**<0.05**
Discharge to other than home	1.18	**<0.05**
Older age	0.99	**<0.05**
Risk Factors for 90-Day Mortality	HR	*P*
CCI score	1.46	**<0.01**
End-stage renal disease (ESRD)	3.62	**<0.05**
AKI	4.92	**<0.01**
Older age	1.05	**<0.01**

Bold text highlights statistical significance.

## Discussion

In this retrospective cohort study using a large national database of hospital readmissions, we found that patients with UC who were readmitted within 30 or 90 days from their index hospitalization had a significantly higher mortality rate (over 4-fold higher) compared to that of their index hospitalization. Additionally, patients with UC had a significantly longer hospitalization and increased hospitalization costs on readmission. We also identified risk factors for 30- and 90-day readmission and mortality. Opioid use, chronic steroid use, VTE, congestive heart failure, hospitalization at a teaching hospital, and a higher number of comorbidities were identified as characteristics that were independently associated with an increased 30-day readmission, while opioid use, anxiety, hospitalization at a teaching hospital, discharge to a facility other than home, and higher number of comorbidities were identified as characteristics independently associated with increased 30-day mortality.

The finding that readmission in UC patients is a predictor for mortality is a relatively novel finding. Prior studies evaluating hospital readmissions for UC have not described in-hospital mortality on readmission, though the risk of death in patients who are readmitted (not restricted to only in-hospital death) has previously been described. A recent study by Kruger et al found that patients who were readmitted within 30 days after an index hospitalization for UC had a 4.5% calendar year mortality rate, compared with 0.45% in those not readmitted within 30 days.^15^ This finding has also been reported in other medical conditions; a study looking at mortality after readmission for heart failure found that the risk of death among readmitted patients within 30 and 90 days was almost 3- and 4-fold higher, respectively.^16^ A hypothesis for this association may be that patients who are readmitted are more likely to have severe disease or associated complications that are associated with a higher risk of death.

With over one-fourth of patients requiring readmission within 90 days of their index hospitalization, hospital readmissions for patients with a primary UC diagnosis represent a significant burden on the healthcare system. Prior single-center studies have reported a 30-day readmission rate between 11.7% and 14% and 90-day readmission rate between 20.5% and 24% for patients with IBD (both UC and Crohn disease), which is similar to our findings.^7,17,18^ However, a study using the 2013 U.S. NRD found a 30-day readmission rate of 10.6%.^19^ While this number is significantly lower than the readmission rate of 17.38% in our study, we hypothesize that some of this discrepancy may be due to differences arising from the use of the older (ICD-9) codes, rather than the newer (ICD-10) codes used in our study.

In our study, the variable with the strongest correlation with 30-day mortality was VTE. Similar to prior studies, we found that patients hospitalized for a UC flare with a history of VTE were noted to have a higher risk of 30-day readmission and 30-day mortality.^20,21^ This has previously been well described, as patients with IBD have an increased risk of VTE, and patients with active disease are more likely to have VTE.^22,23^ Furthermore, patients with IBD are more likely to develop postdischarge VTE than non-IBD patients.^24^

Pain control is an important aspect in the care of patients with IBD, with prior studies demonstrating that chronic pain is associated with an increased risk of readmission.^17^ A study by Hazratjee et al found that patients with IBD who were discharged without opioid analgesia were 2.2-fold more likely to be readmitted within 30 days.^25^ However, opioid use disorder has been associated with an increased risk of 30-day readmission.^26^ In our study, we found that chronic opioid use was a significant predictor of both 30- and 90-day readmission and 30-day mortality. Indeed, it appears that pain control is important, but care must be taken to prevent chronic opiate dependence.^27^ Reja et al found that 2.6% of patients with UC were readmitted within 1 year for opiate use disorder; the mean total charge for readmission for opiate use disorder was $41,291 and associated with a mortality of 0.57%.^28^

Corticosteroids, while very effective for the short-term treatment of IBD, have also been associated with a significant risk of complications, especially when used in the long term.^29^ In fact, prior studies have found that past use of steroids is associated with a higher risk of readmission (odds ratio 1.90).^30^ Our study is consistent with this finding; we found the chronic use of steroids to be a significant predictor of 30-day readmission. However, we note that the short-term use of steroids was not evaluated in this study, and our findings cannot be generalized to the short-term use of steroids. Furthermore, it is unclear whether the higher rate of readmission for patients with steroid use is due to the use of steroids (and their associated adverse effects) or that steroid use is simply a surrogate for more aggressive and refractory disease. Regardless, steroids have been demonstrated to play an important role in the management of IBD flares and its use during index admission can decrease the risk of 30-day readmission.^18^

We also found that anxiety was associated with 90-day readmission and 30-day mortality. Psychiatric comorbidities have previously been noted to increase the risk of readmission in many medical conditions, including IBD.^17,31^ However, the finding of increased risk of 30-day mortality is a little more puzzling. While it is unlikely that anxiety itself increases the risk of death, we hypothesize that anxiety may be associated with having other comorbidities, which we found to be associated with a higher risk of death. Anxiety has also been demonstrated to be associated with disease activity,^32^ though disease severity may not always correlate with mortality.^6^

There are several strengths to our study. First, the use of a large national database provided the ability to draw conclusions that we believe are representative of the US population. Our study also looked at factors associated with inpatient mortality, which we believe has greater clinical significance than readmission rate alone. Furthermore, we looked at both 30- and 90-day readmission and mortality rate, which allows one to determine the persistence of each predictor over time.

We also acknowledge several limitations of our study. Although our study draws its strength from a large sample of patients, it relies on administrative codes which have the potential for misclassification of IBD diagnoses and misrepresentation of reason for hospital admission. A prior study on the use of ICD-9 codes found that ICD-9 codes have low accuracy when it comes to identifying IBD patients.^13^ While the accuracy of ICD-10 codes, which were used in this study, have not yet been studied, we reviewed previously published manuscripts on the topic to confidently include the most appropriate ICD codes. The NRD also does not record race or ethnicity, variables which have been previously demonstrated to play a role in the risk of hospital readmission; black patients with IBD have previously been found to have an increased risk for readmission.^18,33^ Also, our study is not able to measure what happens between hospitalizations, including the risk of death outside of the hospital and the impact of postdischarge follow-up, both of which would provide greater context to the results of this study. Finally, we acknowledge the fact that mortality upon readmission in many patients was due to other causes (other than the UC). However, this is unlikely to effect the overall results for several reasons. First, we only included the UC as a primary diagnosis, and yet over 30% were readmitted for UC. Second, other readmission diagnoses, such as sepsis, were likely to be (at least in some cases) a direct result of the UC. Third, upon sensitivity analysis, our results demonstrate that UC readmissions had higher mortality, as compared to all other readmissions. Hence, we conclude that UC upon readmission is associated with mortality and believed to be one of the major factors driving the increased mortality and hospital LOS/cost found.

In summary, our study highlights that patients with UC have a high rate of hospital readmission and a high rate of mortality, particularly upon readmission. We have also identified critical risk factors that may help decrease the risk of readmission and mortality, as well as reduce healthcare costs, in this important patient population.

## Supplementary Material

otab029_suppl_Supplementary_Table_S1

## Data Availability

The data underlying this article are available in the article and in its online supplementary material.

## References

[1] Pola S , Patel D, Ramamoorthy S, et al. Strategies for the care of adults hospitalized for active ulcerative colitis. Clin Gastroenterol Hepatol. 2012;10:1315–1325.e4. 22835577 10.1016/j.cgh.2012.07.006 PMC4226798

[2] Ng SC , Shi HY, Hamidi N, et al. Worldwide incidence and prevalence of inflammatory bowel disease in the 21st century: a systematic review of population-based studies. Lancet. 2017;390:2769–2778. 29050646 10.1016/S0140-6736(17)32448-0

[3] Bernstein CN , Longobardi T, Finlayson G, et al. Direct medical cost of managing IBD patients: a Canadian population-based study. Inflamm Bowel Dis. 2012;18:1498–1508. 22109958 10.1002/ibd.21878

[4] Kappelman MD , Rifas-Shiman SL, Porter CQ, et al. Direct health care costs of Crohn’s disease and ulcerative colitis in US children and adults. Gastroenterology. 2008;135:1907–1913. 18854185 10.1053/j.gastro.2008.09.012 PMC2613430

[5] Bewtra M , Kaiser LM, TenHave T, et al. Crohn’s disease and ulcerative colitis are associated with elevated standardized mortality ratios: a meta-analysis. Inflamm Bowel Dis. 2013;19:599–613. 23388544 10.1097/MIB.0b013e31827f27ae PMC3755276

[6] Falvey J , Greenwood R, Creed TJ, et al. Mortality in ulcerative colitis—what should we tell our patients? Three year mortality following admission for the treatment of ulcerative colitis: a 6 year retrospective case review. Frontline Gastroenterol. 2010;1:35–41. 28839541 10.1136/fg.2009.000216 PMC5517156

[7] Tinsley A , Naymagon S, Mathers B, et al. Early readmission in patients hospitalized for ulcerative colitis: incidence and risk factors. Scand J Gastroenterol. 2015;50:1103–1109. 25866237 10.3109/00365521.2015.1020862

[8] Hanzlik TP , Tevis SE, Suwanabol PA, et al. Characterizing readmission in ulcerative colitis patients undergoing restorative proctocolectomy. J Gastrointest Surg. 2015;19:564–569. 25560185 10.1007/s11605-014-2734-7 PMC4565166

[9] Nguyen GC , Bollegala N, Chong CA. Factors associated with readmissions and outcomes of patients hospitalized for inflammatory bowel disease. Clin Gastroenterol Hepatol. 2014;12:1897–1904.e1. 24681074 10.1016/j.cgh.2014.02.042

[10] Feuerstein JD , Jiang ZG, Belkin E, et al. Surgery for ulcerative colitis is associated with a high rate of readmissions at 30 days. Inflamm Bowel Dis. 2015;21:2130–2136. 26020605 10.1097/MIB.0000000000000473

[11] Bernstein CN , Nabalamba A. Hospitalization, surgery, and readmission rates of IBD in Canada: a population-based study. Am J Gastroenterol. 2006;101:110–118. 16405542 10.1111/j.1572-0241.2006.00330.x

[12] Sharma S , Weissman S, Mehta TI, et al. Role of hospital teaching status on outcomes of patients with inflammatory bowel disease: a nationwide analysis. Dig Dis Sci. 2021;66:2216–2226. doi:10.1007/s10620-020-06497-8. PMID: 32696235. PMC8385679 32696235

[13] Hou JK , Tan M, Stidham RW, et al. Accuracy of diagnostic codes for identifying patients with ulcerative colitis and Crohn’s disease in the Veterans Affairs Health Care System. Dig Dis Sci. 2014;59:2406–2410. 24817338 10.1007/s10620-014-3174-7 PMC6907154

[14] Stepaniuk P , Bernstein CN, Nugent Z, et al. Characterization of inflammatory bowel disease in elderly hospitalized patients in a large central Canadian Health region. Can J Gastroenterol Hepatol. 2015;29:274–278. 25874650 10.1155/2015/724359 PMC4467489

[15] Kruger AJ , Hinton A, Afzali A. Index severity score and early readmission predicts increased mortality in ulcerative colitis patients. Inflamm Bowel Dis. 2019;25:894–901. 30247551 10.1093/ibd/izy297

[16] Benuzillo JG , Rasmusson KD, Kfoury AG, et al. Mortality after readmission among heart failure patients. J Heart Lung Transplant. 2014;33:S272.

[17] Barnes EL , Kochar B, Long MD, et al. Modifiable risk factors for hospital readmission among patients with inflammatory bowel disease in a nationwide database. Inflamm Bowel Dis. 2017;23:875–881. 28426473 10.1097/MIB.0000000000001121 PMC5512697

[18] Mudireddy P , Scott F, Feathers A, et al. Inflammatory bowel disease: predictors and causes of early and late hospital readmissions. Inflamm Bowel Dis. 2017;23:1832–1839. 28858068 10.1097/MIB.0000000000001242

[19] Poojary P , Saha A, Chauhan K, et al. Predictors of hospital readmissions for ulcerative colitis in the United States: a national database study. Inflamm Bowel Dis. 2017;23:347–356. 28221246 10.1097/MIB.0000000000001041 PMC6071407

[20] Nguyen GC , Sam J. Rising prevalence of venous thromboembolism and its impact on mortality among hospitalized inflammatory bowel disease patients. Am J Gastroenterol. 2008;103:2272–2280. 18684186 10.1111/j.1572-0241.2008.02052.x

[21] Doshi R , Doshi S, Desai J, et al. Trends in hospitalization and in-hospital mortality with inflammatory bowel disease complicated by venous thromboembolism. JGPLD. 2017;4:1–8.29516037

[22] Bernstein CN , Blanchard JF, Houston DS, et al. The incidence of deep venous thrombosis and pulmonary embolism among patients with inflammatory bowel disease: a population-based cohort study. Thromb Haemost. 2001;85:430–434. 11307809

[23] Grainge MJ , West J, Card TR. Venous thromboembolism during active disease and remission in inflammatory bowel disease: a cohort study. Lancet. 2010;375:657–663. 20149425 10.1016/S0140-6736(09)61963-2

[24] McCurdy JD , Kuenzig ME, Smith G, et al. Risk of venous thromboembolism after hospital discharge in patients with inflammatory bowel disease: a population-based study. Inflamm Bowel Dis. 2020;26:1761–1768. 31995204 10.1093/ibd/izaa002

[25] Hazratjee N , Agito M, Lopez R, et al. Hospital readmissions in patients with inflammatory bowel disease. Am J Gastroenterol. 2013;108:1024–1032. 23820989 10.1038/ajg.2012.343

[26] Charilaou P , Mohapatra S, Joshi T, et al. Opioid use disorder increases 30-day readmission risk in inflammatory bowel disease hospitalizations: a nationwide matched analysis. J Crohns Colitis. 2020;14:636–645. 31804682 10.1093/ecco-jcc/jjz198

[27] Cohen-Mekelburg S , Rosenblatt R, Gold S, et al. The impact of opioid epidemic trends on hospitalised inflammatory bowel disease patients. J Crohns Colitis. 2018;12:1030–1035. 29741667 10.1093/ecco-jcc/jjy062 PMC6113704

[28] Reja M , Hajela N, Makar M, et al. One-year risk of opioid use disorder after index hospitalization for inflammatory bowel disease. Int J Colorectal Dis. 2020;35:2081–2087. 32681379 10.1007/s00384-020-03691-y

[29] Waljee AK , Wiitala WL, Govani S, et al. Corticosteroid use and complications in a US inflammatory bowel disease cohort. PLoS One. 2016;11:e0158017. 27336296 10.1371/journal.pone.0158017 PMC4918923

[30] Christian KE , Jambaulikar GD, Hagan MN, et al. Predictors of early readmission in hospitalized patients with inflammatory bowel disease. Inflamm Bowel Dis. 2017;23:1891–1897. 28837523 10.1097/MIB.0000000000001213

[31] Ahmedani BK , Solberg LI, Copeland LA, et al. Psychiatric comorbidity and 30-day readmissions after hospitalization for heart failure, AMI, and pneumonia. Psychiatr Serv. 2014;66:134–140. 25642610 10.1176/appi.ps.201300518 PMC4315504

[32] Byrne G , Rosenfeld G, Leung Y, et al. Prevalence of anxiety and depression in patients with inflammatory bowel disease. Can J Gastroenterol Hepatol. 2017;2017:e6496727. https://www.hindawi.com/journals/cjgh/2017/6496727/ (12 December 2020, date last accessed). 10.1155/2017/6496727 PMC5664260 29181373

[33] Gunnells DJ Jr , Morris MS, DeRussy A, et al. Racial disparities in readmissions for patients with inflammatory bowel disease (IBD) after colorectal surgery. J Gastrointest Surg. 2016;20:985–993. 26743885 10.1007/s11605-015-3068-9

