# Supplementary material for: Increased Mortality and Healthcare Costs Upon Hospital Readmissions of Ulcerative Colitis Flares: A Large Population-Based Cohort Study
Source: Crohns Colitis 360. 2021 Jun 9;3(3):otab029. doi: 10.1093/crocol/otab029 (PMC9802231; doi:10.1093/crocol/otab029)
Supplement: otab029_suppl_Supplementary_Table_S1 [file otab029_suppl_supplementary_table_s1.docx]

**Supplementary Table 1.** The International Classification of Diseases, 10th revision (ICD-10) codes used to generate results.

| Anxiety | F41.1; F41.3;  F41.8; F41.0;  F41.9; F41;  F40.8; F43.22 |
| --- | --- |
| Acute kidney injury (AKI) | N17.0; N17.1; N17.2; N17.8; N17.9 |
| Congestive heart failure (CHF) | I50.XXX |
| Chronic steroid use | Z79.52 |
| End-stage renal disease (ESRD) | N186; Z992; I120; I1311; I132; I120 |
| Opioid use | F11.XX |
| Ulcerative colitis (UC) | K51.90, K51.91 |
| Venous thromboembolism (VTE) | I82.XX, I26.XX |
